# Image-Guided Treatment of Primary Liver Cancer in Mice Leads to Vascular Disruption and Increased Drug Penetration

**DOI:** 10.3389/fphar.2020.584344

**Published:** 2020-09-30

**Authors:** Sara B. Keller, Dingjie Suo, Yak-Nam Wang, Heidi Kenerson, Raymond S. Yeung, Michalakis A. Averkiou

**Affiliations:** ^1^Department of Bioengineering, University of Washington, Seattle, WA, United States; ^2^Applied Physics Laboratory, University of Washington, Seattle, WA, United States; ^3^Department of Surgery, University of Washington, Seattle, WA, United States

**Keywords:** ultrasound, drug delivery, vascular disruption, microbubbles, hepatocellular carcinoma, doxorubicin

## Abstract

Despite advances in interventional procedures and chemotherapeutic drug development, hepatocellular carcinoma (HCC) is still the fourth leading cause of cancer-related deaths worldwide with a <30% 5-year survival rate. This poor prognosis can be attributed to the fact that HCC most commonly occurs in patients with pre-existing liver conditions, rendering many treatment options too aggressive. Patient survival rates could be improved by a more targeted approach. Ultrasound-induced cavitation can provide a means for overcoming traditional barriers defining drug uptake. The goal of this work was to evaluate preclinical efficacy of image-guided, cavitation-enabled drug delivery with a clinical ultrasound scanner. To this end, ultrasound conditions (unique from those used in imaging) were designed and implemented on a Philips EPIQ and S5-1 phased array probe to produced focused ultrasound for cavitation treatment. Sonovue^®^ microbubbles which are clinically approved as an ultrasound contrast agent were used for both imaging and cavitation treatment. A genetically engineered mouse model was bred and used as a physiologically relevant preclinical analog to human HCC. It was observed that image-guided and targeted microbubble cavitation resulted in selective disruption of the tumor blood flow and enhanced doxorubicin uptake and penetration. Histology results indicate that no gross morphological damage occurred as a result of this process. The combination of these effects may be exploited to treat HCC and other challenging malignancies and could be implemented with currently available ultrasound scanners and reagents.

## Introduction

Hepatocellular carcinoma (HCC) is the sixth most common cancer globally and the fourth leading cause of cancer-related deaths (El-Serag and Rudolph, 2007; Global Burden of Disease Liver Cancer Collaboration et al., 2017). The high case mortality rate can be attributed to the delay in diagnosis, lack of effective systemic therapy, and pre-existing liver disease that limits hepatic reserve. The major risk factors for HCC are the same as those causing liver cirrhosis including alcoholism, hepatitis B/C, and steatohepatitis. Consequently, treatment options and prognosis depend not only on tumor characteristics, but also the extent of liver dysfunction, rendering many strategies too aggressive. Emerging therapies have focused on more precise tumor targeting while sparing non-tumor liver (Mavros et al., 2014; Liang et al., 2016).

One such approach is the use of vascular disruption agents (VDAs) to restrict blood flow to the tumor (Liu et al., 2017). However, small-molecule VDAs such as combrestatin A-4 phosphate (CA4P) and 5, 6-dimethylxanthenone-4-acetic acid (DMXAA) are often associated with poor side effects and can result in drug resistance in some cases (Liang et al., 2016; Gill et al., 2019). Ultrasound-mediated microbubble cavitation has been shown to recapitulate the vascular disruptive effect with more precise targeting, thereby limiting side effects (Wang et al., 2015). The immature neovessels within angiogenic tumors are abnormal, with nonuniform vessel diameters, irregular branching, and heterogeneous blood flow patterns (Fukumura and Jain, 2007). It is hypothesized that these aberrant architectures make the vasculature of tumors more vulnerable to the mechanical effects of cavitation than surrounding non-tumor tissue. Therefore, it has been shown that cavitation-induced vascular disruption can be selectively applied to the tumor microvascular network, while avoiding damage to surrounding tissue (Wang et al., 2015). Cavitation activity within the irregular tumor microvasculature causes selective vascular shutdown, hypothesized to be a result of a decrease in microvascular density due to widespread endothelial cell toxicity and vascular depletion (Wang et al., 2015). Indeed, several studies have shown significant blood flow restriction to tumor neovessels with cavitating microbubbles at modest ultrasound pressures (1–5 MPa) with minimal non-tumorous tissue damage. Goertz et al. showed significant reduction of blood flow in the central area of tumors after therapy with 1.65 MPa at a frequency of 1 MHz (Goertz et al., 2012). In a study performed by Wang et. al, microvascular density was significantly reduced 24 h after therapy with pressures beyond 1.5 MPa, with no damage to surrounding tissue until the sonication pressure reached 5 MPa (Wang et al., 2015). Finally, Liu et al. observed complete cessation of tumor blood flow after therapy with 4.8 MPa that could last as long as 24 h, which resulted in widespread necrosis to the tumor region (Liu et al., 2012).

Despite these successes, tumor hypoxia as a sole strategy for cancer treatment can often have the unintended consequence of an increased proliferative phenotype in remaining tumor cells and endothelial cells (Jain, 2005; Liu et al., 2017; Gill et al., 2019). Moreover, prior work suggests that the effect of vascular shutdown may be offset by an opposing “vascular rebound,” since the cancer cells that remain after therapy tend to be more aggressive, often resulting in worse clinical outcomes (Goertz et al., 2012; Liu et al., 2017). Therefore, a synergistic approach to targeting both the aberrant neovessels in the tumor core through cavitation-mediated vascular disruption and the proliferative outer rim of the tumor through enhanced cytotoxic drug penetration may be necessary to overcome these limitations (Siemann et al., 2002; Goertz et al., 2012).

Previous studies that have examined ultrasound-mediated vascular disruption with or without drug delivery *in vivo* have generally used simplistic single element transducers for the ultrasound treatment which limits clinical translation (Goertz et al., 2012; Ho et al., 2018; D’Souza et al., 2019). Single element focused transducer systems provide a great deal of flexibility for parameter optimization, but they are cumbersome to use due to requiring auxiliary components (function generators, amplifiers, etc.) and they are not capable of imaging. In addition, the use of custom devices in clinical trials requires regulatory approval, which is a complex process. Because of this, there has been a clear trend from single element transducers towards clinical imaging transducers for ultrasound therapy. However, the lack of ability to modify scanner pulsing parameters has limited innovation in this area. Indeed, in a Phase I clinical trial using ultrasound and microbubbles to enhance gemcitabine delivery to pancreatic cancer (Dimcevski et al., 2016), the investigators chose to use normal imaging modes with short sound pulses for therapy, despite the fact that their previous studies indicated that “sonoporation had a significant therapeutic effect when using long pulse durations.” It can be inferred that the authors of the reported study were unable to create the acoustic parameters necessary for maximally effective therapy. More recently, Mason et al. reported using a Philips X5-1 for interleaved imaging and therapy for tissue perfusion augmentation (Mason et al., 2020). While the application of that work was not targeted drug delivery and therefore wide beam areas were preferred, it still represents an important example of the adaptation of clinical ultrasound technology for interventional procedures.

The main objective of the present work is to evaluate preclinical efficacy of image-guided, cavitation-induced vascular disruption and drug uptake enhancement using a clinical ultrasound scanner in a physiologically relevant mouse model of primary liver cancer, defined in the present manuscript as UltraSound Cavitation Treatment (USCTx). Our hypothesis is that using a clinical diagnostic probe to perform USCTx along with clinical microbubbles will result in vascular disruption and greater doxorubicin delivery in treated tumors. To the best of our knowledge, we believe that this is the first study to incorporate clinically available tools and reagents in a pre-clinical *in vivo* model of genetically engineered mice to highlight the combined effects of vascular disruption and drug delivery. If successful, this would be a simple, inexpensive, and easily implementable clinical technique for treating human liver tumors.

## Materials and Methods

### Breeding of Pten-Null Mouse Model

All animal work was conducted in accordance with national guidelines and was approved by the Institutional Animal Care and Use Committee (IACUC) at the University of Washington, Seattle. 8 week old male albumin (Alb)-Cre mice (003574, B6.Cg-Tg(Alb-cre)21Mgn/J) and 8-week old female Pten^fl/fl^ mice (006440, B6.129S4-Ptentm1Hwu/J) were purchased from Jackson Laboratory (Bar Harbor, ME). Alb-Cre mice were bred with Pten^fl/fl^ mice to ultimately generate Pten^fl/fl^;Alb^cre^ experimental mice. Genotyping protocols and primers were obtained from Jackson Laboratory, and PCR was performed to confirm the correct genotype. At 40 weeks of age, Pten^fl/fl^;Alb^cre^ (*Pten*-null) mice develop tumors that are physiologically similar to human HCCs and ICCs (intrahepatic cholangiocarcinoma) (Horie et al., 2004; Kenerson et al., 2011). Moreover, the mice develop hepatic steatosis, resulting in livers that are abnormally large preceding tumorigenesis. Tumor progression was monitored through weekly ultrasound scans with an L15-7io imaging probe on a Philips iU22 (Philips Medical Systems, Bothell, WA) starting at 36 weeks of age. Mice (20 male and 18 female) were treated once tumors were 1 cm in diameter.

### Drug and Contrast Agents

Doxorubicin was used as the chemotherapeutic drug as it is detectable using fluorescent imaging. Although clinically, the liposomal formulation of doxorubicin, Doxil, is favored over the free drug, we chose to use free doxorubicin for simplicity, and note that it could be easily replaced by a wide variety of other small molecule drugs. Furthermore, the benefits of using Doxil over doxorubicin (ie, limiting cardiotoxicity) would not be relevant in the current study due to the acute timeline of the experiments. Doxorubicin HCl was purchased and dissolved in sterile saline at a concentration of 10 mg/ml and mice received a dosing level of 30 mg/kg (Li et al., 2015). Sonovue^®^, marketed in the US as Lumason^®^ (Bracco Suisse SA, Geneva, Switzerland), was the contrast agent used for these studies and was resuspended according to the manufacturer’s instructions. Microbubble concentration was measured using a Multisizer 3 (Beckman Coulter, Brea, CA, USA) and found to consistently be between 1 and 5 × 10^8^ microbubbles/ml. Microbubble dosing was evaluated through preliminary experiments in which it was observed that 50-μl injections gave adequate contrast without acoustic shadowing (Keller et al., 2019) and therefore that dosing regimen was used for all further experiments.

### Acoustic Parameters

Focused ultrasound beams suitable for USCTx were designed and implemented on a Philips EPIQ scanner and S5-1 phased array (Philips Healthcare, Bothell, WA, USA) operating in a hybrid pulsed-wave Doppler mode. A deep focal length of 10 cm was chosen to allow for a broader collimated beam in the nearfield (1–2 cm) where the liver tumor in the mice would be. The scanner was modified to produce 200 cycles at a pulse repetition frequency (PRF) of 50 Hz at acoustic pressures ranging between 2 and 3 MPa (Mannaris and Averkiou, 2012) at a center frequency of 1.6 MHz. The output pressure could be easily modified by changing the transmit voltage to the probe in the same fashion as done during clinical scanning. The specific ultrasound parameters were selected to be consistent with prior work studying cavitation induced treatments with ultrasound and microbubbles (Goertz et al., 2012; Liu et al., 2012; Wang et al., 2015). The spatial extent of the sound field in both the azimuth and elevation planes at the chosen transmit voltage (100 V) were measured in a water tank with a 0.4 mm membrane hydrophone (Precision Acoustics Ltd., Dorchester, UK), acquired using a DPO7054C Oscilloscope (Tektronix, Inc., Beaverton, OR, USA) and analyzed in MATLAB (The MathWorks, Inc., Natick, MA, USA). The delivered acoustic pressure in vivo may be calculated by derating the water measurements according to the acoustic attenuation in tissue.

### Treatment Procedure

**Figure 1:**
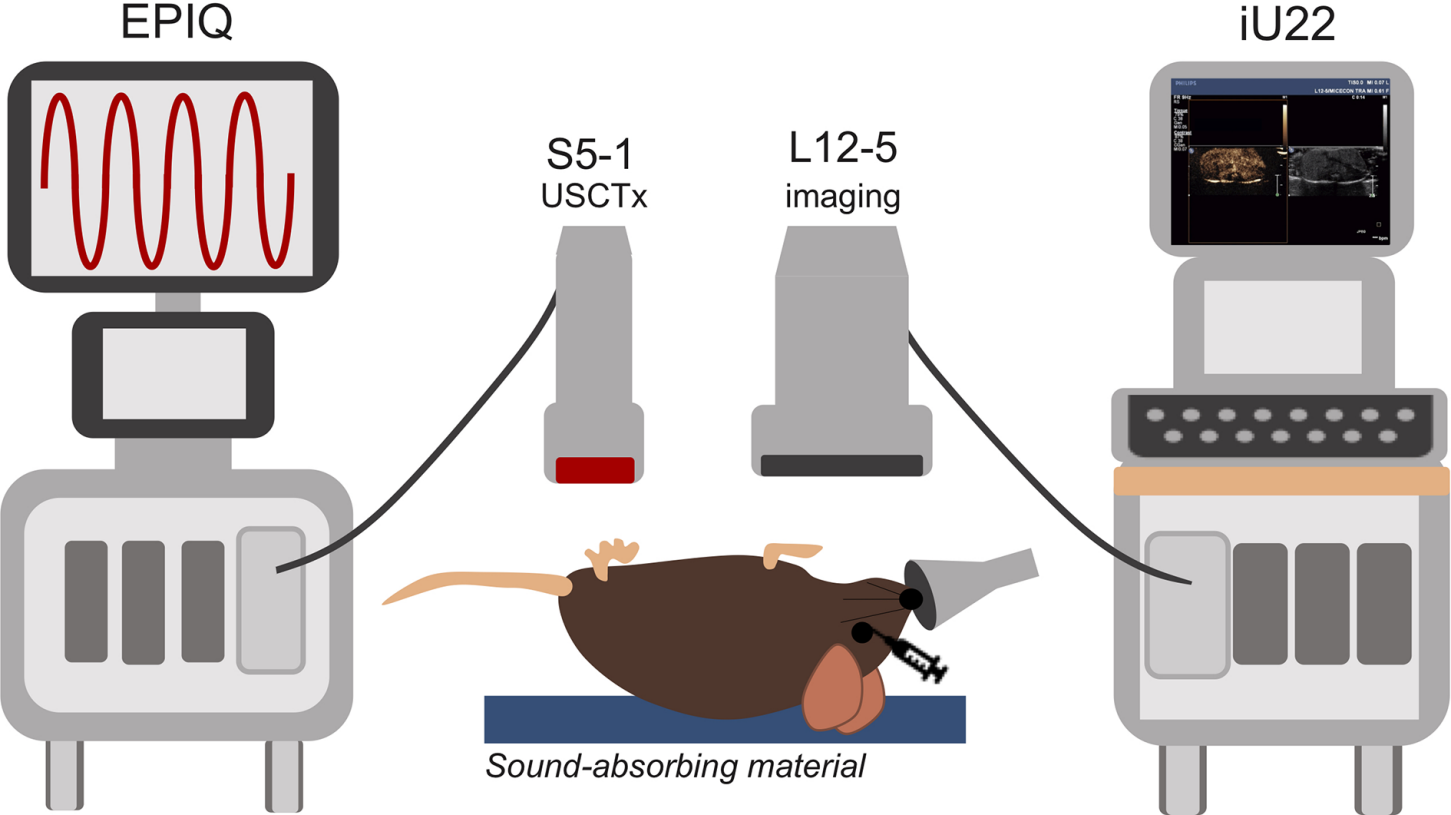
Overview of experimental setup. The EPIQ and S5-1 probe were programmed to produce ultrasound conditions for cavitation treatments. The iU22 with the L12-5 probe were used for contrast enhanced ultrasound (CEUS) imaging before and after cavitation treatments as well as routine monitoring of tumor growth. Mice were placed on a sound-absorbing material to minimize sound reverberations.

**Figure 2:**
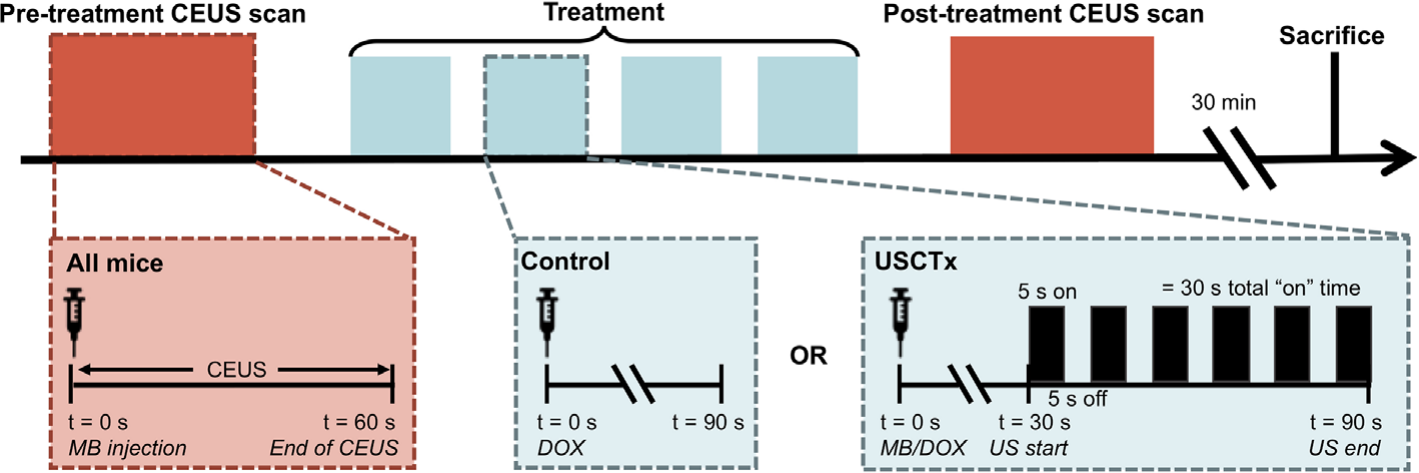
Overview of experimental protocol. 60 s CEUS loops were taken before and after therapy on all mice, regardless of experimental group. Treatment is defined by 4 injections of DOX alone (control) or DOX + USCTx. There were 90 s between each injection. The mice were sacrificed 30 min after the post-treatment CEUS exam.

**Figure 3:**
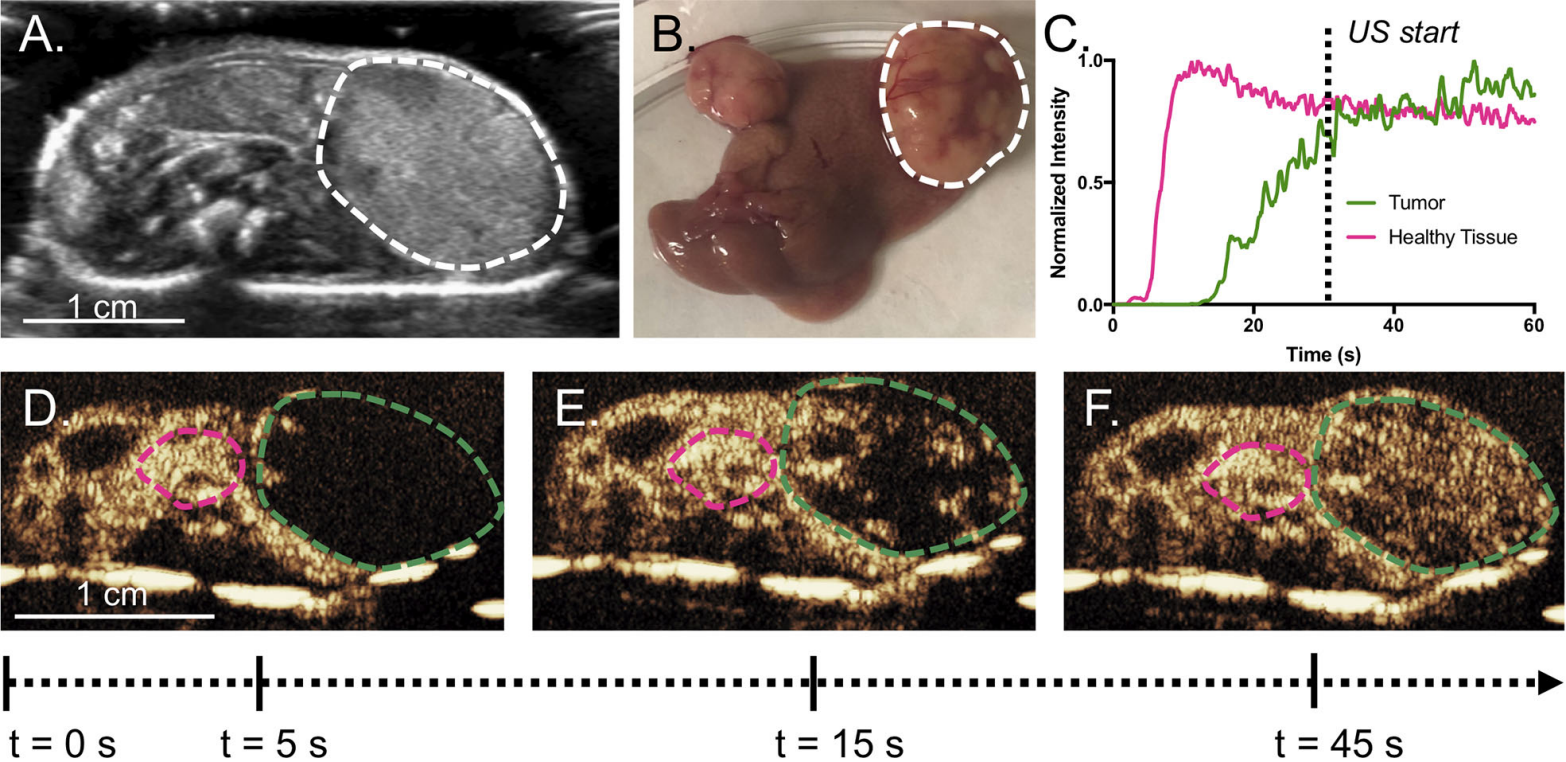
**(A)** Representative cross-sectional B-mode image of a mouse with a tumor (bounded by dashed line) taken with the L12-5. Tumors can be easily delineated by gross anatomical morphology, as in **(B)**. Example TICs from the tumor and the normal parenchyma are shown in **(C)** with **(D–F)** showing the original CEUS images and ROIs, where the green line is the tumor and the pink line is healthy tissue. Intensity was normalized to peak intensity within each respective ROI, in order to highlight differences in time parameters. In this example, the tumor does not reach its peak intensity until after the 60 s. The wash-in of microbubbles is slower in the tumor than the surrounding tissue. USCTx was chosen to start at 30 s, when the tumor had sufficient microbubble accumulation.

Mice were anesthetized under 1% to 3% isoflurane and placed supine on a sound-absorbing pad to minimize sound reverberations (**Figure 1**). After depilating the abdomen of the mouse, pre-treatment b-mode images were taken to orient the imaging plane in the center of the tumor. An overview of the experimental timeline may be seen in **Figure 2**. Two ultrasound scanners, one for USCTx (EPIQ) and one for imaging (iU22) were used. A pre-treatment contrast-enhanced ultrasound (CEUS) scan was performed with a Philips iU22 and L12-5 linear array probe on all mice. 50 μl Sonovue^®^ was injected retro-orbitally and 60 s CEUS loops were recorded. The L12-5 was then removed and the S5-1 probe of the EPIQ with programmed long pulses (described in the previous section) was positioned over the same area. This timing also allowed for clearance of microbubbles from pre-treatment CEUS imaging. Treatment consisted of doxorubicin (DOX) with or without USCTx. Mice were randomly split into focused ultrasound (DOX + USCTx) or control (DOX alone) cohorts. There were 10 male and 9 female mice in each group. Mice receiving USCTx received 4 injections of DOX + MBs over one anesthesia event. Each injection was followed by focused ultrasound treatment 30 s after injection which alternated between “on” for 5 s and “off” for 5 s (Keravnou et al., 2016), for a total “on” time of 30 s. The start time of 30 s was chosen based on evaluating bolus transit in preliminary contrast injections (Averkiou et al., 2019), and the multiple injection regimen was chosen to maximize the time in which ultrasound affected microbubbles at peak bolus concentrations (Keravnou et al., 2016). Time intensity curve (TIC) analysis (Dietrich et al., 2012) was used to determine the optimal time for treatment (**Figure 3**). Control animals received doxorubicin alone without USCTx. The total doxorubicin amount in control animals was divided into 4 injections with a 90 s waiting period between injections to mimic the timing of ultrasound in the USCTx cohort. 30 min after the last injection, mice were euthanized *via* transcardial perfusion and samples of tumor and healthy liver were embedded in optimum cutting temperature (OCT) compound for sectioning. Tumor versus non-tumor tissue could be delineated based on gross anatomical morphology after dissection (**Figure 3B**). Half of the tumor tissue was frozen for sectioning. The other half of the tumor tissue, along with non-tumorous liver parenchyma, heart, kidney, spleen, and muscle were removed and frozen at −80°C for doxorubicin extraction.

### CEUS Evaluation of Vascular Disruption

CEUS pre- and post- treatment video loops [extracted as native DICOM data (Dietrich et al., 2012)] were analyzed qualitatively for image intensity in tumor and non-tumor liver areas, as well as quantitatively using TIC analysis in QLAB (Philips Healthcare, Bothell, WA, USA). Contrast agent rise time (RT), which is inversely proportional to blood flow (Strouthos et al., 2010), and peak intensity (PI), which is a measure of vascular volume (D’Souza et al., 2019), were measured as indicators of perfusion changes (Keravnou et al., 2016). Regions of interest (ROIs) were drawn as freeform polygons in tumor and non-tumor areas on the contrast image. The intensity within an ROI is the average intensity of the pixels over the entire area. RT was calculated as the time it took from microbubbles arriving in each ROI (tumor and non-tumor) to the peak of contrast intensity. Change in RT (ΔRT) is expressed as the post-treatment RT minus the pre-treatment RT and therefore higher values of ΔRT would indicate a slower perfusion rate in the ROI as a result of treatment. PI within the tumoral area was calculated as the maximum contrast intensity within the ROI and was normalized to an ROI of surrounding non-tumor tissue intensity in order to account for differences in microbubble injections. Change in PI is expressed as a ratio of the post-treatment PI divided by the pre-treatment PI and therefore a value less than 1 would indicate a reduced PI in the ROI as a result of treatment. Since the intensity within the non-tumor region was used for normalization, changes in non-tumor PI were not evaluated. Perfusion parameters from 25 (11 control and 14 treated) tumors were analyzed. Loops from mice that had inadequate bubble delivery and/or had obvious differences in probe placement pre- and post- treatment were not analyzed for perfusion changes due to potential for inaccuracies.

### Quantification of Doxorubicin Uptake

Quantification of doxorubicin uptake was performed as described previously (Laginha et al., 2005). Briefly, nuclear lysis (RIPA) buffer (50 mM Tris•HCl, 150 mM NaCl, 1% Triton X-100, 0.5% Sodium Deoxycholate, 0.1% SDS, 1mM EDTA, 10 mM NaF, 1 mM PMSF, in ddH_2_0) was added to frozen tissue samples (20% w/v) in 2 ml centrifuge tubes and homogenized on ice with hard tissue homogenizer tips (Omni International, Kennesaw, GA, USA). 200 μl of tissue homogenate was removed and added to a 2-ml microcentrifuge tube, along with 100 μl 10% v/v Triton X-100, 200 μl ddH_2_O, and 1,500 μl 0.75 N acidified isopropanol. Samples were vortexed and then left at −20°C for overnight extraction. The next day, samples were warmed to room temperature, vortexed, and centrifuged at 15,000*g* for 20 min. The supernatant was added to a 96 well plate and analyzed for fluorescence intensity (480 nm Ex; 605 nm Em) using a plate reader (Infinite 200 PRO, Tecan, Austria). Fluorescence was compared against a standard curve of known absolute doxorubicin amounts added to untreated tissue homogenate. Doxorubicin amount is presented in micrograms (per equivalent tissue homogenate mass). Samples were omitted if the tumor or organ volume was insufficient to get an accurate reading.

### Fluorescence Microscopy and Histologic Examination

Serial sections of 5 μm thickness were taken from OCT-embedded tumor and liver samples from all mice using a Leica CM 1950 Cryostat (Leica Biosystems). At up to three different locations near the central region of the tumor, one section was analyzed qualitatively for doxorubicin presence using a custom filter set (480/40 nm Ex; 605/50 nm Em; dichroic, 505 lp). The second section was stained with anti-CD31 (Abcam, Cambridge, United Kingdom) for evaluation of microvascular integrity. The final section was stained with hematoxylin and eosin (H&E) for anatomical morphology, which enabled segregation of tumor and non-tumor tissue, and was used to evaluate gross morphological damage. CD31 and H&E staining were performed by the UW Histology and Imaging Core.

### Statistical Analysis

Doxorubicin uptake between control and USCTx treated groups was compared using a Mann-Whitney test. Change in RT and post-PI divided by pre-PI were compared between control and USCTx treated groups using an unpaired Student’s *t*-test. Statistical analyses were performed in GraphPad Prism version 7.0 for Mac (GraphPad Software, San Diego, CA, USA). For all analyses, the significance level was set to 0.05.

## Results

### Measured Acoustic Sound Field From Clinical Device

**Figure 4:**
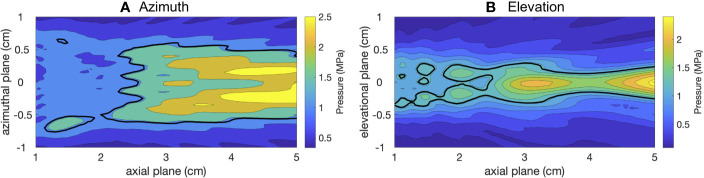
2D sound fields of the therapeutic beam from the S5-1 in azimuth **(A)** and elevation **(B)** planes.

Hydrophone measurements of the ultrasound pressure field in the azimuthal and elevational planes in water of the S5-1 clinical probe (used for USCTx) can be seen in **Figures 4A, B**, respectively. The black line represents the −6 dB contours of the maximum spatial extent (area of up to half the maximum amplitude). The azimuthal beam width (**Figure 4A**) is about 1 cm in diameter and reaches pressures up to 2.5 MPa (in this specific amplitude setting). The elevational beam width (**Figure 4B**) is slightly narrower (0.5 cm). Given that mice were treated once their tumors reached approximately 1 cm in diameter, it can be extrapolated that the beam covered essentially the entire tumor area. Despite the fact that a diagnostic ultrasound device was used to generate these beams, it can be seen that the amplitude is slightly higher than the FDA limit of 1.9 MI given the transmit frequency of 1.6 MHz. Since these measurements are in water, to find the actual *in situ* pressure in the mice we must adjust for attenuation at a 0.3 dB/(cm-MHz) (Keravnou C. P. et al., 2015). Since the tumors were generally located within 2 cm of the transducer face, attenuation was minimal (approximately 0.96 dB, corresponding to a loss of 10% of the transmitted pressure). We note that the probe is in a hybrid PW Doppler mode and the number of cycles and acoustic pressure are not what would be used in standard Doppler imaging.

### Vascular Disruption Measured by Qualitative and Quantitative CEUS

**Figure 5:**
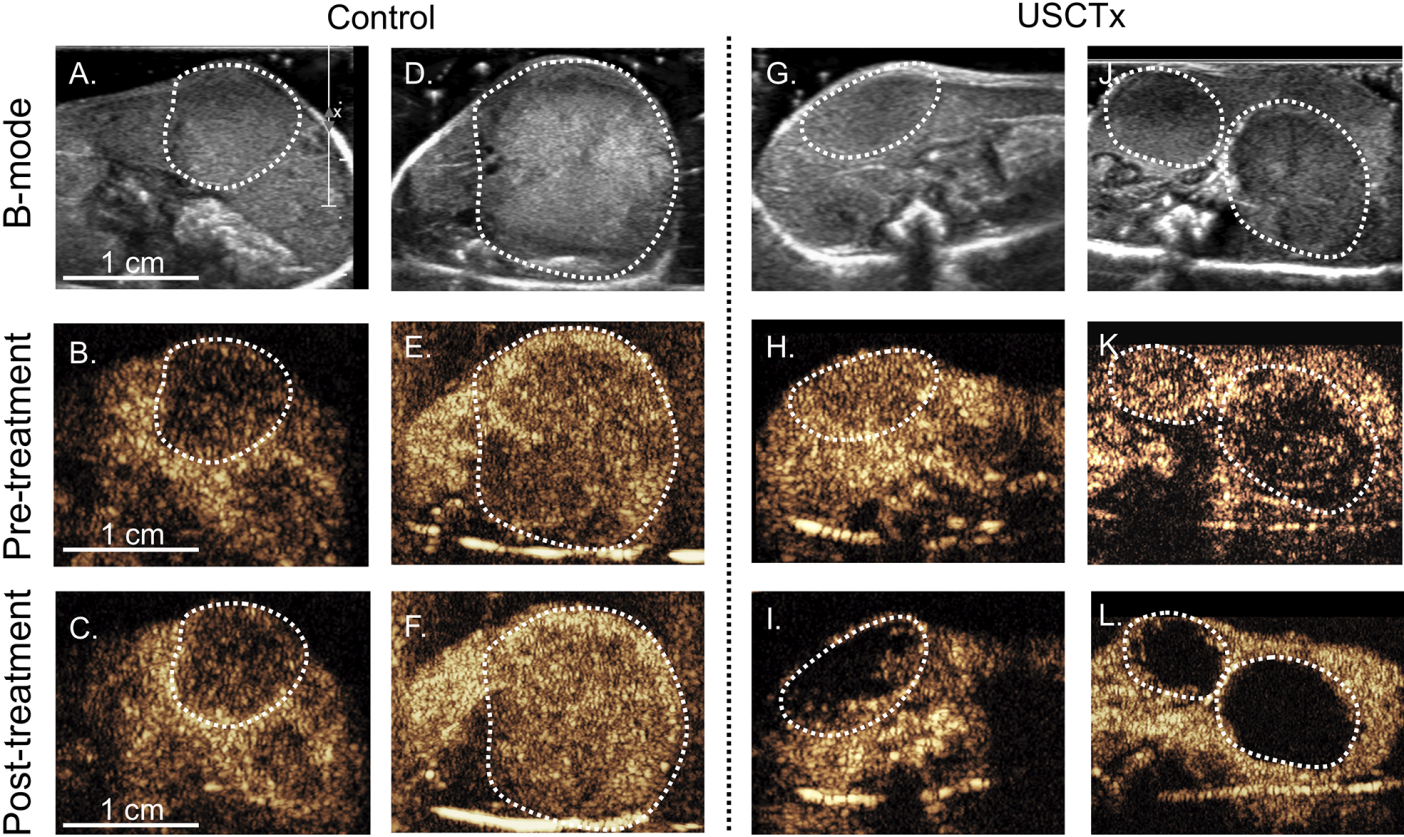
Each column represents four different mice with tumors indicated by the dashed white line; **(A–F)** did not undergo USCTx while **(G–L)** did undergo USCTx. The top row **(A, D, G, J)** shows the initial b-mode images, the middle row **(B, E, H, K)** shows CEUS images taken 15 s after microbubble injection before treatment, and the bottom row **(C, F, I, L)** shows CEUS images taken 15 s after microbubble injection after treatment. Note reduced presence of microbubbles post-treatment in the USCTx mice **(I, L)** and similarities in microbubble presence in the control mice **(C, F)**.

**Figure 5** shows CEUS images from pre- and post-treatment loops. The top row (**Figures 5A, D, G, J**) shows the anatomical b-mode image, and the middle (**Figures 5B, E, H, K**) and bottom (**Figures 5C, F, I, L**) rows show the contrast images acquired 15 s after a bolus injection before treatment and after treatment, respectively. There was no reduction in contrast in the tumors shown in the control mice post-therapy (**Figures 5C, F**). However, for mice treated with USCTx, areas of perfusion deficits may be seen in (**Figures 5I, L**). Any differences in the overall image brightness is attributed to differences in the actual microbubble concentration delivered with the bolus injection.

**Figure 6:**
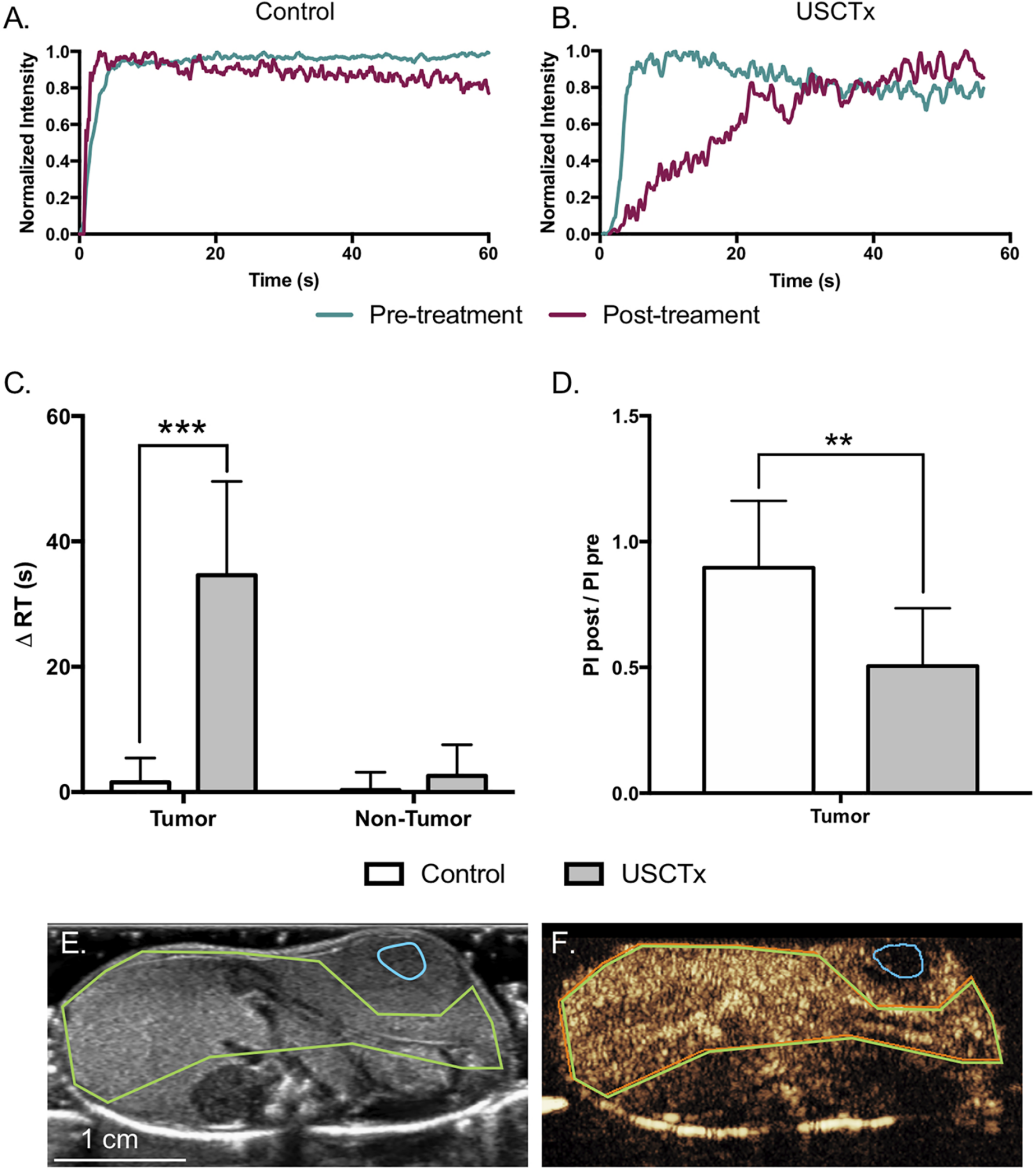
Representative TICs from control **(A)** and USCTx **(B)** mice within the tumor. Signal intensity was normalized to the maximum intensity in each individual ROI in order to better visually demonstrate change in time parameters as in **Figure 3**. Quantification of ΔRT in control and USCTx mice in the tumor and non-tumor liver is shown in **(C)**. Post-treatment PI divided by pre-treatment PI within the tumor is shown in **(D)**. ROIs showing the tumor (blue) and non-tumor tissue (green) is shown in b-mode **(E)** and contrast **(F)**. **p < 0.01, ***p < 0.001.

**Figure 6** shows quantitative analysis of CEUS loops. Representative tumor TICs from control and USCTx mice are shown in **Figures 6A, B**, respectively. Signal intensity in these representative TICs were normalized to the maximum intensity in each individual ROI (such that the maximum intensity in each TIC equals 1) in order to better visually demonstrate the time it took from the beginning of the bolus to the peak (defined as RT), and it can be seen that the RT in the control mouse remained around 5 s, whereas the RT increased to over 40 s in the USCTx mouse. Summary data can be seen in **Figures 6C, D**, where USCTx caused a significant increase in ΔRT within the tumor (p < 0.001). This means that USCTx, on average, increased RT by 34.6 s. There were no significant differences in ΔRT seen in non-tumor (surrounding liver) tissue from USCTx treated mice as well as tumor and non-tumor tissue from control mice. USCTx also resulted in significant reduction in tumor PI (p < 0.01). Example ROIs may be seen in **Figures 6E, F**, where the tumor is bounded in blue and the non-tumor area in green.

### Qualitative and Quantitative Doxorubicin Uptake

**Figure 7:**
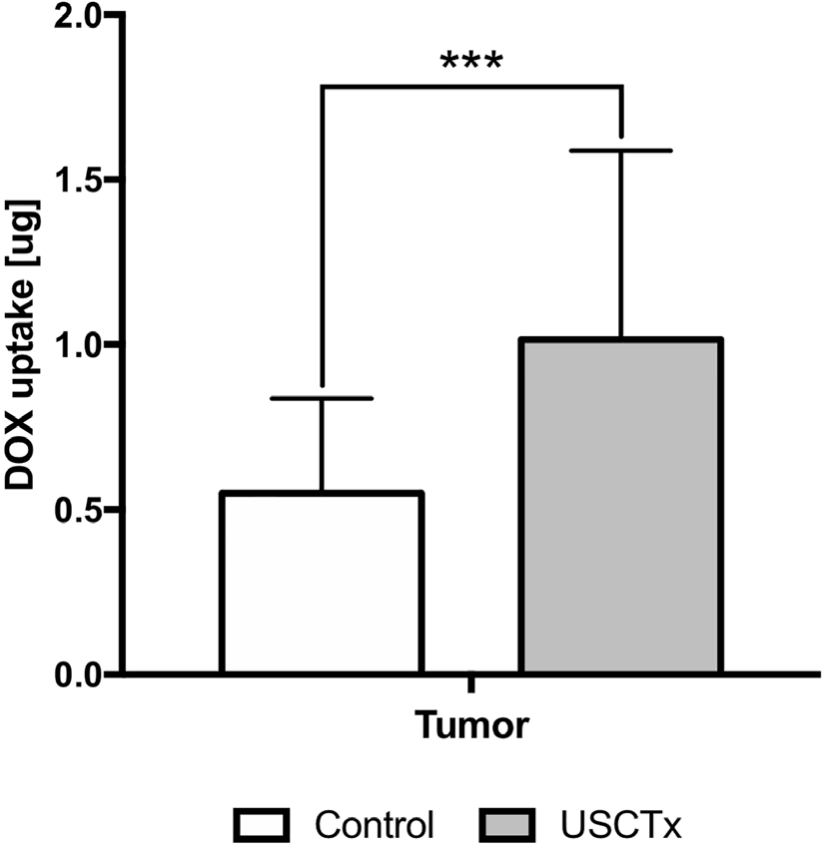
DOX uptake in USCTx versus control mice. Doxorubicin amount is presented in micrograms (per equivalent tissue homogenate mass). ***p < 0.001.

**Figure 7** shows quantification of doxorubicin uptake by fluorescence analysis. Doxorubicin amount is presented in micrograms (per equivalent tissue homogenate mass). There was a significant (p < 0.001), almost twofold, increase in doxorubicin amount in the tumor following USCTx. We also observed significant increases in doxorubicin in other organs (p < 0.01 for spleen, p < 0.05 for liver, heart, muscle and kidney) from USCTx versus control mice.

**Figure 8:**
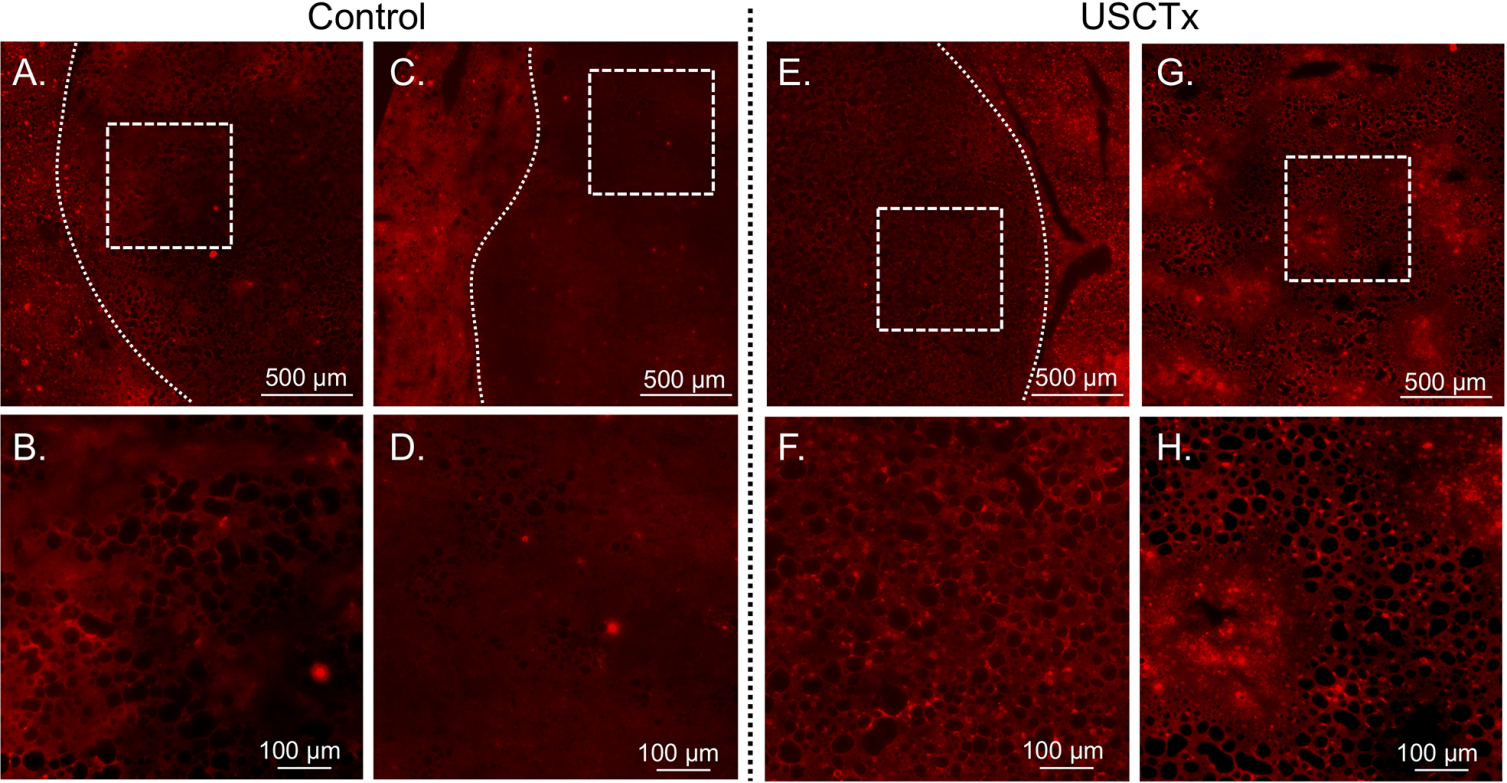
Representative DOX fluorescent images may be seen from control animals in **(A–D)** and USCTx animals in **(E–H)**. Each column represents a different animal. The top row **(A, C, E, G)** shows tumor morphology (tumor bounded with dashed line, or the entire image is the central part of the tumor) and the bottom row **(B, D, F, H)** shows the same area at increased magnification. The USCTx animals have more DOX-positive nuclei than the control animals, as seen in **(F, H)** as compared to **(B, D)**.

A comparison of representative sections taken for DOX nuclear uptake is shown in **Figure 8**. It can be observed that there is a higher accumulation of doxorubicin beyond tumor walls in USCTx versus control animals, as indicated by the greater amount of red fluorescent cell nuclei shown in **Figures 8F, H** than in **Figures 8B, D**.

### Fluorescence Microscopy and Histologic Examination

**Figure 9:**
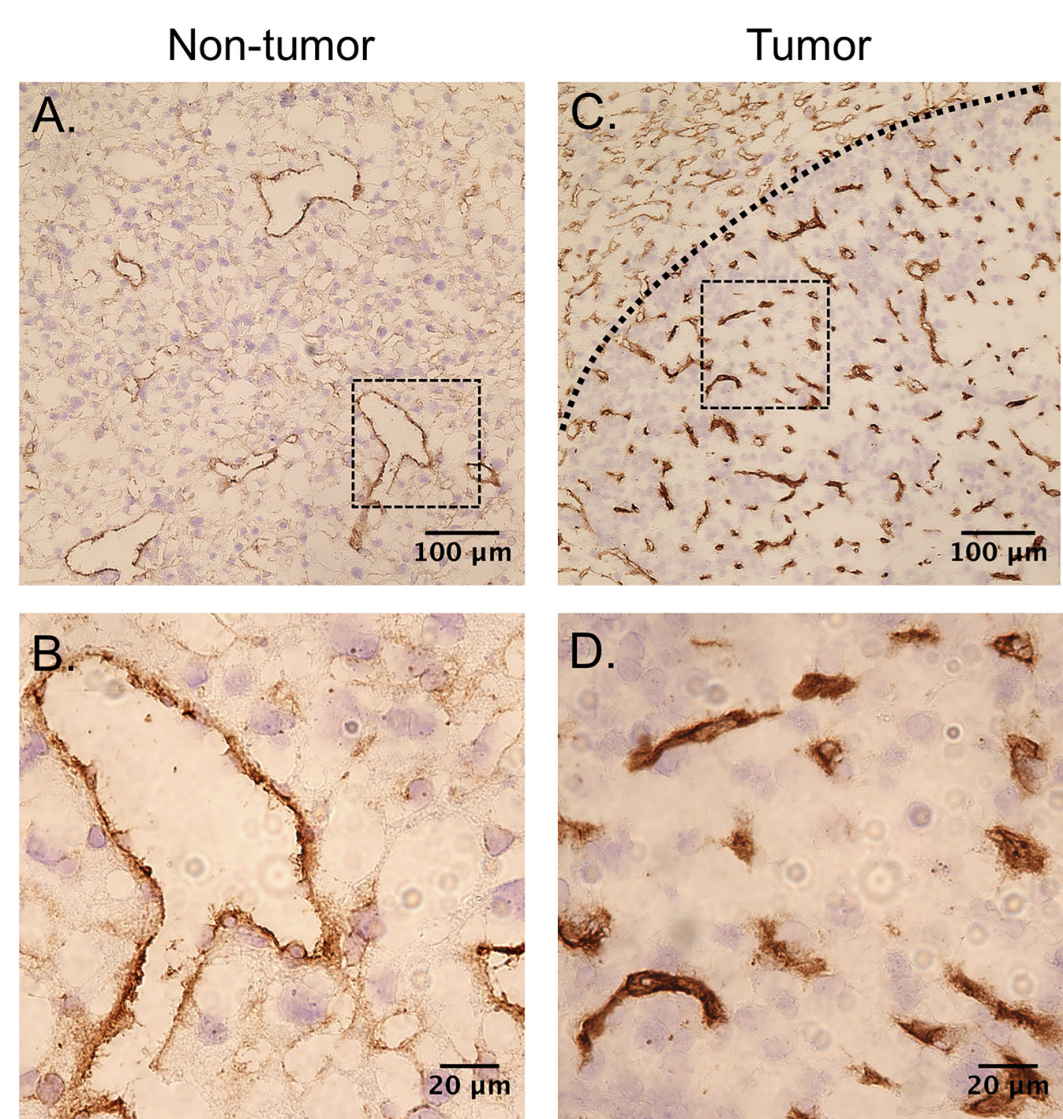
CD31-stained slices can be seen from **(A, B)** non-tumor tissue and **(C, D)** tumor tissue. The vessels in both tumors are much smaller in diameter and the overall vascular density is higher than in the non-tumor section.

CD31 images taken from non-tumor liver tissue and tumor tissue are shown in **Figures 9A, B** and **Figures 9C, D**, respectively. The vascular density of the tumors is much higher than the surrounding tissue. The capillaries in the tumors were also in general smaller in diameter than the non-tumor liver. No differences in microvascular density were observed between USCTx and control animals.

**Figure 10:**
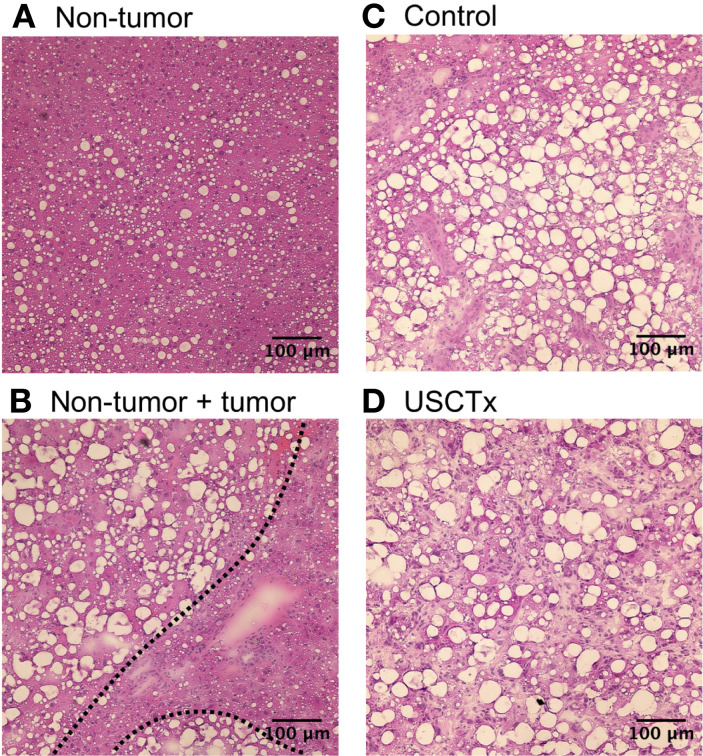
Examples of H&E stained images from non-tumor tissue **(A)**, the boundary between non-tumor and tumor tissue **(B)**, control tumorous tissue **(C)** and USCTx tumorous tissue **(D)**. No tissue damage was observed in any slides.

Representative examples of H&E stained sections are shown in **Figure 10**. Both non-tumor tissue and tumor tissue had some degree of steatosis. It can be observed that it was simple to segment tumorous tissue from non-tumorous tissue using anatomical morphology in **Figure 10B**. No hemorrhaging or extravasated red blood cells were observed in any H&E stained slides in either USCTx or control animals (**Figures 10C, D**).

## Discussion

The main goal of the present work was to investigate ultrasound cavitation-induced treatment (USCTx) of primary liver cancer while paying attention to the ability of the present methods to be easily translated to clinical practice. To this end, we used a genetically engineered mouse model that developed native liver cancer that could appropriately recapitulate human pathology and we performed all imaging and treatment with clinical ultrasound systems. Enhanced drug penetration and immediate tumor vascular shutdown were observed in animals treated with focused ultrasound and microbubbles compared to those receiving free drug. Using a diagnostic ultrasound device, clinical-grade microbubbles, and a clinically approved drug means that this technique could be adopted into clinical practice.

### Use of a Diagnostic Ultrasound Device for Ultrasound Cavitation Treatment

To the best of our knowledge, this is the first study to optimize a clinical scanner and probe to produce the ultrasound conditions for cavitation treatment that are different from those of normal imaging. We chose the S5-1 array for two reasons: it allows the use of a low frequency (1.6 MHz was the lowest available for this probe) suitable for cavitation therapies and it produces collimated beams to cover a large area, like novel phased array HIFU devices currently being investigated (Hand et al., 2009; Wang and Zhou, 2016; Aslani et al., 2019). We chose to implement parameters that were consistent with other studies investigating cavitation-induced vascular disruption, and therefore the MI of 2 that was used was only slightly higher than the FDA limit of 1.9. It is worth noting, however, that our cycle lengths are slightly longer than would typically be used within these FDA constraints. Optimizing ultrasound treatment parameters in this regime requires changing aspects of the Doppler signal path, such as its depth (focus), sample volume size and range (number of cycles and PRF), and mechanical index (focal pressure). The highest sample volume achievable with the S5-1 without additional modification yields 32 cycles, but thermal index limitations on the probe means that the mechanical index at this setting is limited to 0.5. By accessing research settings on the system, we were able to increase the number of cycles and pressure, while simultaneously decreasing PRF and reducing the thermal load on the probe. While a higher number of cycles could be used, there is a tradeoff between output voltage, number of cycles, and PRF, due to concerns of transducer surface heating. That said, there is still a high degree of tunability that can be achieved within these constraints. Finally, although the S5-1 is technically also able to perform imaging, we chose to instead use a higher frequency linear probe for better image resolution in mice. However, if this technique were to be used in clinical abdominal applications, the operating frequency of the S5-1 would be suitable to perform both treatment and imaging, and two scanners would not necessarily be required.

### Pten-Null Mice Are an Appropriate Model for Studying Image-Guided Cavitation Treatment

In addition to using a diagnostic ultrasound device for treatment, we also chose to use a mouse model that could appropriately mimic human liver cancer. Despite previous reports showing efficacy in ultrasound-mediated vascular disruption with or without drug delivery (Wood et al., 2005; Goertz et al., 2012; Liu et al., 2012; D’Souza et al., 2019), there have been no studies using a physiologically relevant preclinical liver cancer model. However, one drawback to using this model is the substantial time to tumorigenesis (40 weeks in addition to breeding time). While subcutaneous or intramuscular tumor inoculations provide quicker experimental timelines, it was important to study these phenomena in a system that more accurately represents the *in vivo* setting of liver cancer.

From H&E stained slides, large fat vacuoles can be seen due to the steatotic nature of the *Pten*-null phenotype. Steatosis is associated with an overall reduction in perfusion in the hepatic microcirculation, due to the enlargement of parenchymal cells from fat accumulation causing distortion of liver sinusoids (Farrell et al., 2008). This, in turn, can yield less efficient drug delivery (Ito et al., 2006). In addition to steatosis, two forms of primary liver cancer, HCCs and ICCs, were observed in the livers of *Pten*-null mice. HCC accounts for 70% of diagnosed liver cancer cases and ICC accounts for 15% (Massarweh and El-Serag, 2017). ICC often accompanies HCC during clinical diagnosis and generally has the same risk factors, including cirrhosis, hepatitis B/C, and non-alcoholic steatohepatitis (Massarweh and El-Serag, 2017). Therefore, despite increasing the complexity of the model, the presence of both forms of primary liver cancer (HCC and ICC) along with steatohepatitis is not uncommon in disease progression in humans.

### Microbubble-Mediated Vascular Shutdown

We observed that microbubble cavitation at moderate ultrasound pressures between 2 and 3 MPa exhibits a high degree of anti-vascular action selectively within the tumors of mice. This was first seen qualitatively, in which it was observed that there was a clear reduction of contrast in the tumor post-USCTx that was not observed in healthy tissue. When this was analyzed quantitatively using TIC analysis, we found a significant increase in contrast RT and a significant decrease in PI in the tumor cores of USCTx mice. No differences in RT and PI were observed in non-tumor tissue of either control or USCTx treated mice, consistent with prior studies showing selectivity of cavitation treatment within the tumor (Goertz et al., 2012; Wang et al., 2015). This selectivity is generally hypothesized to be a result of the fragile and atypical vascular structure in tumor neovessels, including irregular vessel diameters and branching patterns (Semela and Dufour, 2004; Fukumura and Jain, 2007). Prior studies have shown these highly aberrant vessels are more susceptible to cavitation-induced damage, including endothelial cell damage and thrombosis (Hwang et al., 2005; Wang et al., 2015). Differences in vascular morphology were confirmed through CD31 staining of endothelial cells; as shown in **Figure 9**, the vascular density of tumor tissue was indeed higher than in non-tumorous tissue. Moreover, the vessel size was much smaller in the tumors, averaging about 10 to 20 µm, as opposed to the non-tumor vessels, which had diameters in the order of hundreds of microns. Given that microbubbles are within the 1- to 10-µm range, it makes sense that cavitation within these smaller diameter vessels might have a bigger impact on blood flow. However, no observable differences in CD31 expression were seen in USCTx versus control animals in both tumorous and non-tumorous tissue, nor was any damage observed in any H&E stained slides, indicating that no acute endothelial damage occurred. However, this may be due to the fact that microvascular density changes are generally observed after a latency period of 24 h after therapy (Wang et al., 2015). Alternatively, the exact mechanism may be due to other physiological changes (such as modulating tumor interstitial pressure), and the lack of gross morphological damage may be an indicator of the overall safety of this therapy.

### Enhanced Drug Penetration in Ultrasound Cavitation-Treated Tumors

We found that there was a significant, almost twofold, increase in doxorubicin accumulation in the tumors of USCTx mice versus control mice. When other organs were considered, however, we also observed significant increases. This might be due to the fact that we used a broad sound beam and, though we tried to minimize sound reflections through the use of an attenuating pad, our setup may still have allowed for other organs to have been affected. This could be easily avoided in clinical scenarios by image guidance and beamforming approaches for targeting of the therapeutic ultrasound field to be strictly within the tumoral area. Indeed, the size of the S5-1 makes it more optimized for targeting in human liver HCCs rather than in mice. The quantitative observations were further confirmed using qualitative fluorescence imaging of doxorubicin presence in serial sections of frozen tissue. It was observed that there was on average more penetration of doxorubicin deeper into the tumors of USCTx versus control animals. Attaining high tumor volumes usually comes at the expense of high dosages, which can lead to cardiotoxicity among other dangerous side effects (Singal and Iliskovic, 1998). Therefore, achieving higher doxorubicin accumulation in the tumor with the same injected dose is of high value, and this technique could certainly be implemented with other current drugs like sorafenib (Tan et al., 2019) or even nucleic acids (Scarabel et al., 2017; Di Ianni et al., 2019).

There is some discrepancy between the observed reduction in perfusion (and therefore reduction in blood flow) and contradictory enhanced drug delivery. One possible explanation for this is that the enhanced permeability and retention (EPR) effect seen in many highly angiogenic tumors can result in a high interstitial fluid pressure that can compromise drug delivery (Semela and Dufour, 2004). One proposed mechanism of how ultrasound and microbubbles increase drug penetration in tumors is that cavitation activity actually temporarily alleviates the tumor interstitial fluid pressure, allowing for enhanced drug diffusion (Zhang et al., 2019). Most likely is that the two aspects of USCTx, drug penetration and anti-vascular action, work synergistically to target the outer rim and core of the tumor, respectively (Goertz et al., 2012). However, a longer-term study would be required to confirm this hypothesis.

### Limitations

The purpose of this study was to investigate the feasibility of performing ultrasound-mediated cavitation treatment *in vivo* using a clinical ultrasound scanner. That being said, there are still many interesting mechanistic questions that were beyond the scope of that goal. Future work will be focused on exploring the exact physiological mechanism and time duration of vascular disruption in addition to long-term survival. However, the acute nature of these experiments allowed us to see immediate tissue morphology and drug accumulation both histologically and quantitatively as a result of USCTx. Additionally, we focused only on one set of ultrasound parameters. We specifically selected parameters based on previous work from our lab; 200 cycles and 50 Hz PRF to maximize cavitation activity and minimize microbubble motion (Mannaris and Averkiou, 2012; Keller et al., 2019) and 2 to 3 MPa for maximum bubble response (Keravnou C. et al., 2015). These parameters remain consistent with published literature (Goertz et al., 2012; Wang et al., 2015); however, more parameter optimization may be necessary to study the underlying physical and physiological mechanisms of USCTx and would be simple to perform with the S5-1. Finally, this animal model represents a rather specific form of liver cancer that develops from a single genetic mutation. Unsurprisingly, the typical pathogenesis of HCC is far more complex, often a result of repeated stress on the liver due to chronic diseases such as Hepatitis B/C and alcoholic cirrhosis causing eventual DNA damage. Yet, HCC prevalence rates are rising in Western countries, which is theorized to be a result of an increase in obesity and obesity-related illnesses, such as diabetes, which cause hepatic steatosis and eventually liver cancer (Starley et al., 2010). In this case, the *Pten*-null mouse model may be considered a reasonable model for observing macroscopic perfusion and drug delivery in cases in which cancer develops in the liver in the presence of steatosis.

## Conclusion

A diagnostic ultrasound scanner was converted into a device capable of performing ultrasound cavitation treatment by reprogramming the transmit sequence of the S5-1 probe in a hybrid PW Doppler mode. The *Pten*-null mouse model used in these studies exhibited steatosis in addition to HCC and ICC tumors, consistent with some types of human liver cancer pathogenesis. We observed that there were significant and immediate perfusion changes following ultrasound cavitation treatment with a commercial scanner and microbubbles. This effect was specific to the tumor, implying an inherent vulnerability in the tumor microvasculature that was not seen in surrounding tissue. Furthermore, it was shown that there was a statistically significant increase of doxorubicin in tumors in treated versus control animals. We did not observe any gross morphological damage, nor any differences in the microvasculature of USCTx treated tumors. The combination of selective tumor vascular disruption and peripheral enhanced drug penetration may play a synergistic role in successful cancer treatment.

## Data Availability Statement

The raw data supporting the conclusions of this article will be made available by the authors, without undue reservation.

## Ethics Statement

The animal study was reviewed and approved by University of Washington Institutional Animal Care and Use Committee.

## Author Contributions

MAA and RSY conceived of the study. SBK, DS, Y-NW, and HK performed the experiments. SBK, Y-NW, and RSY evaluated the histology slides. SBK and MAA wrote the manuscript. All authors contributed to the article and approved the submitted version.

## Funding

This work was supported by a grant from the U.S. Department of Defense (CA160415/PRCRP).

## Conflict of Interest

The authors declare that the research was conducted in the absence of any commercial or financial relationships that could be construed as a potential conflict of interest.
